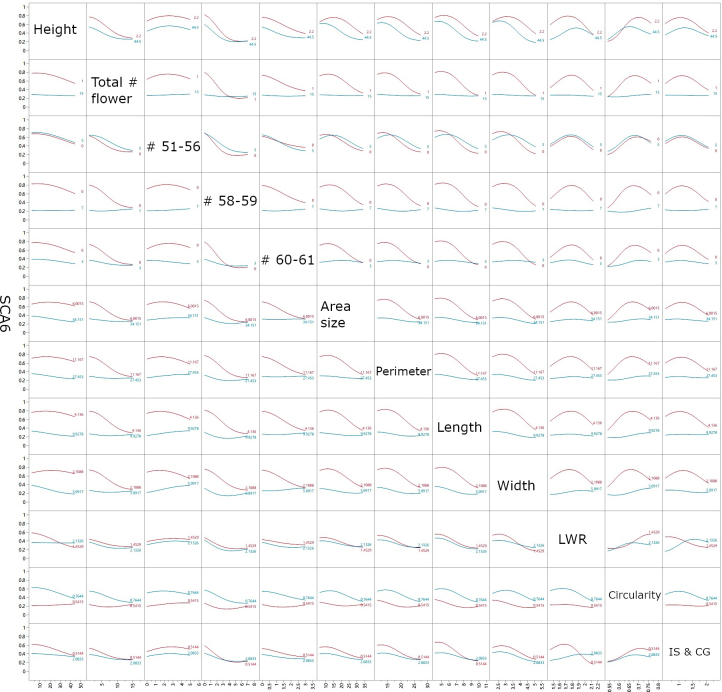# Cacao floral traits are shaped by the interaction of flower position with genotype

**DOI:** 10.1016/j.heliyon.2025.e42407

**Published:** 2025-02-03

**Authors:** Seunghyun Lim, Insuck Baek, Seok Min Hong, Yoonjung Lee, Silvas Kirubakaran, Moon S. Kim, Lyndel W. Meinhardt, Sunchung Park, Ezekiel Ahn

**Affiliations:** a Sustainable Perennial Crops Laboratory, Agricultural Research Service, United States, Department of Agriculture, Beltsville, MD, 20705, USA; b Environmental Microbial and Food Safety Laboratory, Agricultural Research Service, United States, Department of Agriculture, Beltsville, MD, 20705, USA; c Department of Civil Urban Earth and Environmental Engineering, Ulsan National Institute of Science and Technology, UNIST-gil 50, Ulsan, 44919, Republic of Korea; d Department of Plant Pathology, University of Minnesota, Saint Paul, MN, 55108, USA; e Grape Genetics Research Unit, Agricultural Research Service, United States, Department of Agriculture, Geneva, NY, 14456, USA

**Keywords:** Cacao, Flower, Morphology, Shape, Size, Vertical height, Environmental and physiological gradients

## Abstract

Understanding the factors influencing cacao flower morphology and abundance is essential for optimizing productivity. This study investigated the influence of vertical flower position and associated environmental and physiological gradients on these traits across two cacao genotypes, CCN51 and SCA6, under controlled greenhouse conditions. We measured flower size (lateral area, length, width, and perimeter), shape, and abundance at different developmental stages and vertical tree heights. Significant variations were observed between genotypes and across vertical positions, highlighting the roles of genetic and environmental factors in cacao reproductive biology. For example, CCN51 exhibited significantly larger flowers, with an average area of 18.63 mm^2^ compared to 14.88 mm^2^ for SCA6. Leveraging machine learning techniques, particularly Support Vector Machine, we successfully predicted genotypes based on flower measurements with high accuracy, achieving an area under the receiver operating characteristic curve of 0.87. These findings emphasize the phenotypic diversity of cacao flowers and demonstrate the potential of machine learning in genotype identification, offering valuable insights for breeding and cultivation strategies to enhance cacao productivity.

## Highlights

• Cacao flower size and shape vary by genotype and vertical position, reflecting genetic and environmental influences.
• SVM models accurately predict cacao genotypes from flower measurements.
• Vertical position influences flower morphology and abundance.

## Graphical abstract

**Figure:**
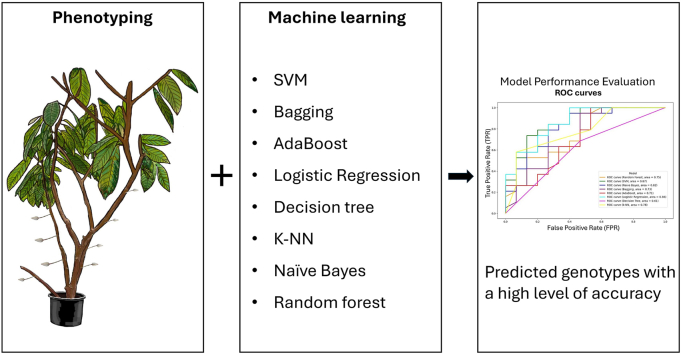

## 1 Introduction

Cultivated worldwide, cacao (*Theobroma cacao* L.) is an important crop that plays an essential role in the global economy and the chocolate industry [1]. As a major crop in tropical regions, cacao production in countries such as Ghana and the Ivory Coast is crucial as it is an export commodity that supports the livelihoods of millions of farmers [2]. Although cacao is of great economic importance, its cultivation is often characterized by low-input and low-output systems that require a deeper understanding of cacao's reproductive biology to improve yields and promote sustainable agriculture [3].

A key aspect of cacao reproductive biology is the flowering process, which transitions the plant from vegetative growth to reproductive maturity throughout lifecycle [4] and significantly influences productivity traits such as anthesis time, stigma receptivity duration, fruit formation, successful pollination, and seed development [5]. Variations in these flowering traits, including the number and qualitative characteristics of flowers, are attributed to both genetic and environmental factors, ultimately impacting cocoa yield [4,6]. A study conducted in Nigeria, for example, compared different cocoa genotypes and revealed significant variations in flowering traits, potentially due to the genetic basis for these differences, particularly in traits like flowering age, which showed a high heritability [6].

Cacao flowering is a complex process influenced by a multitude of factors, including temperature, photoperiod (day length), resource availability (light, water, nutrients), and biotic interactions with pollinators and pathogens [7]. While cacao trees produce an abundance of flowers, only a small fraction of them result in successful pollination and fruit set [8]. This highlights the need for a deeper understanding of the factors that influence flower development and reproductive success. Plants in environments with limited resources, typical of higher latitudes and altitudes with shorter growing seasons, often exhibit adaptive strategies like earlier flowering and reduced flower size [7,9]. This strategic shift ensures reproductive success before the onset of unfavorable conditions. Research on *Chrysanthemum indicum* L. has unveiled that while increased resources can lead to larger plants and more flowers, individual flower sizes diminish under low light conditions, indicating a prioritization of flower quantity over size when resources are abundant [9]. These factors not only determine the timing of flowering but also influence crucial aspects of reproductive success, such as seed size, dispersal mechanisms, and attractiveness to pollinators [7]. The spatial distribution of resources within a plant also significantly influences flower development [10]. The vertical position of flowers within a tree can create environmental and physiological gradients, such as variations in light exposure and hormone distribution, which may affect flower size, shape, and reproductive potential [11]. This study investigates the influence of vertical flower position and associated gradients on flower traits in two cacao genotypes, CCN51 and SCA6, under controlled greenhouse conditions. Cacao's unique cauliflorous structure, with flowers blooming directly from the trunk and main branches, and its hooded anthers complicate pollination [12]. Cacao flowering follows distinct developmental stages [13], from initial bud emergence (stage 51) to full expansion with a color shift (stages 58–59), culminating in bud opening (stages 60–61).

To better understand these complex interactions and their effects on flower traits, this study employs machine learning techniques. Machine learning has emerged as a transformative force in plant biology, offering the potential to unravel complex patterns and relationships within large datasets [[14], [15], [16], [17]]. In the context of cacao, while machine learning has been applied to areas such as fermentation [18], post-harvest management [19], canopy estimation [20], cacao yield [21], and aroma [22], its application in understanding the intricacies of flower development remains unexplored. This study aims to address this gap by comprehensively assessing how flower position and genotype interact to influence key floral traits in cacao, providing a deeper understanding of cacao reproductive biology and informing targeted breeding strategies for optimized pollination and fruit set. We hypothesize that environmental and physiological gradients associated with vertical flower position, in conjunction with genotypic variation, influence flower morphology and abundance in cacao. While this study focuses specifically on cacao, the exploration of how vertical position within the canopy influences floral traits through environmental gradients may have broader relevance to other species. We integrated statistical analysis, bioinformatics, and machine learning techniques to uncover patterns and relationships within the two genotypes of cacao. Through this machine learning-driven approach, we aim to (1) Predict cacao genotypes based on flower measurements, potentially improving breeding and cultivation strategies. (2) Uncover subtle genetic and environmental influences on floral traits, providing a deeper understanding of cacao reproductive biology. (3) Demonstrate the efficacy of various selected machine learning algorithms in predicting key floral traits such as size and shape, aiding the way for its broader application in cacao and other tropical crop research. This study represents an effort to assess the impact of vertical flower position and genotype on floral traits. This work presents the first comprehensive assessment of how flower position and genotype interact to influence key floral traits in cacao, advancing our understanding of cacao floral morphology and emphasizing the potential for targeted breeding strategies to optimize pollination and fruit set.

## 2 Materials and methods

### 2.1 Overview

**Fig. 1:**
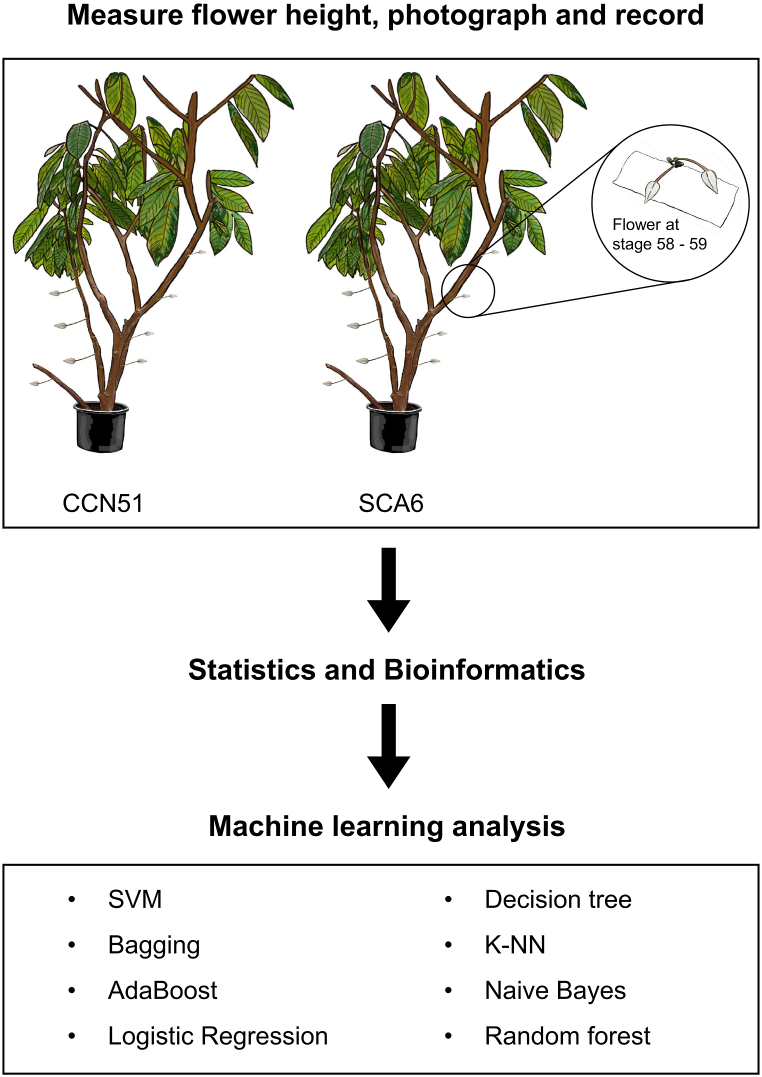
Research Workflow and Methodology. This schematic diagram illustrates the key steps of our research methodology. Phenotypic data was collected from cacao trees, including tree height measurements, photographs, and counts of flower numbers on each cushion. Flower growth stages were recorded, and photographs of flowers at stages 58–59 were analyzed to extract morphological traits. The collected data was analyzed using statistical and bioinformatics tools to identify meaningful patterns. Finally, machine learning algorithms were applied to predict genotypes and understand the genetic basis underlying the observed phenotypic variations.

We conducted a controlled greenhouse experiment using two cacao genotypes (CCN51 and SCA6, as illustrated in Fig. 1) and employed a suite of machine learning algorithms. The popularity of CCN 51 has increased worldwide, especially in Latin America, due to its high productivity, disease resistance, and adaptability to various eco-geographical regions and environments [23]. In contrast, SCA6 is characterized by a high rate of flower bud death and a lower percentage of successful flower opening [6]. We focused specifically on stages 58–59 of flower development when buds are fully expanded but remain closed. This stage was chosen for its distinct elliptical shape in lateral view, which allows for precise measurement before flowering. The vertical location of each flower on each tree and the number of flowers at stages 58–59 and other developmental stages were recorded. This data was stratified by height, and the flower count per cushion (the swollen region on the trunk or branch where cacao flowers emerge) was also noted.

The complexity and high dimensionality of the data collected in this study necessitate machine learning techniques, as traditional statistical methods would likely struggle to identify and interpret the intricate patterns and interactions present. To comprehensively explore the phenotypic space and capture subtle variations, we utilized a diverse toolkit of eight well-established machine learning models, encompassing linear (Logistic Regression, Support vector machine (SVM)-Linear Kernel), non-linear (SVM-Radial basis function kernel (RBF), Decision Tree, Random Forest, k-nearest neighbors algorithm (k-NN), Naive Bayes), and ensemble methods (Bagging, AdaBoost). The selected machine learning algorithms allowed us to comprehensively assess and capture potential patterns within the flower phenotypic data.

### 2.2 Plant materials and greenhouse evaluation

The cacao genotypes CCN51 and SCA6 were evaluated in this study. Clonal plants were originally received from Penn State between 2006 and 2010 and maintained as bentwood plants to encourage chupon growth. Three trees of each genotype, clonally propagated from semi-hardwood stem cuttings, were used for this study in a greenhouse at the USDA-ARS, Beltsville, MD, USA. Cuttings were maintained under propagation mist until the second flush of leaf growth had hardened. Rooted cuttings were transplanted to a soilless media mix of sand:perlite:ProMix BX (2:2:1, v:v:v) and maintained under tropical greenhouse conditions (29-24 °C day/night with 60 % relative humidity) with a 12-h photoperiod (natural daylight supplemented with 400W HID lighting). Plants were fertigated daily with an automatic irrigation/Dosatron system using Peters' peat-lite water-soluble fertilizer (18-8-17 + Magnesium and micronutrients) to ensure a consistent supply of nutrients. The plants were eventually re-potted to 2- or 3-gallon pots with the same soilless media mix and maintained under the above-growing regime. This pot size was chosen to provide adequate space for root development during the experimental period. In August 2024, the vertical location of each flower (measured in cm from the base of the trunk), number of flowers, and developmental stage (earlier than stage 58, stages 58–59, or later/open flowers) on each flower cushion were recorded. Flowers at stages 58–59 were photographed in lateral view with a ruler included in the image for scale to facilitate subsequent analysis of size and morphological traits.

### 2.3 Image analysis

The flowers at stages 58–59 were phenotypically characterized using SmartGrain software (version 1.3) [24]. This software, originally designed for seed morphology, was adapted for our study due to its ability to accurately quantify morphological traits even in non-seed plant structures. Traits measured included area, length, width, length-width ratio (LWR), perimeter, circularity, and the distance between the intersection of length and width (IS) and the center of gravity (CG). The deliberate selection of flowers at stages 58–59 was based on their nearly fully grown state, the noticeable color transition from green to white, and their larger size, giving them the appearance of opened flowers. The elliptical shape of these flowers in lateral view, resembling plant seeds, made them amenable to analysis using SmartGrain software.

### 2.4 Statistical analysis

Phenotypic data comprising twelve traits were collected from CCN51 and SCA6 cacao genotypes. The data were imported into JMP Pro 17 for initial processing. Missing values were handled using Multivariate Normal Imputation [25] with least squares prediction and the shrinkage option to enhance covariance matrix estimation. Two-sided pooled t-tests were conducted to compare phenotypic traits between the two genotypes, and Pearson correlation coefficients were calculated to assess trait relationships within each genotype (Variance estimate = Row-wise, Matrix format = square). Principal Component Analysis (PCA) was conducted to explore the phenotypic diversity and trait relationships (Standardized method, Variance estimate = Row-wise). Additionally, hierarchical clustering was performed on all traits using Ward's linkage & Standardized method in JMP Pro 17 to identify groups of similar traits. To assess the stability of the clustering, bootstrap values for each node were computed with 1,000 replications using the pvclust package (v2.2) in R [26].

### 2.5 Classification algorithms for genotype prediction

We employed a dataset that included flower measurements and corresponding genotypes (CCN51 or SCA6), encompassing size and shape-related traits, flower count at various stages and total per cushion, and vertical flower height. The dataset was split into training (80 %) and testing (20 %) sets using stratified sampling with a fixed random seed for reproducibility. We trained eight classification models on the training set using the scikit-learn library: Random Forest [27], SVM (linear kernel) [28], Naive Bayes [29], Bagging [30], AdaBoost [31], Logistic Regression [32], Decision Tree [33], and k-NN [34]. Hyperparameters for each model were fine-tuned using GridSearchCV with cross-validation. Model performance was assessed on the testing set using metrics such as accuracy, precision, recall, F1-score, and Area Under the Curve (AUC). Receiver Operating Characteristic (ROC) curves were plotted, and feature importance scores were compared across models to identify the most influential genotype predictors.

We also employed SVM with RBF kernel in JMP17 Pro to classify genotypes based on all features. This separate analysis was conducted to leverage JMP's capabilities for in-depth visualization and exploration of feature interactions. We utilized the default settings of Cost = 1 and Gamma = 0.083. A holdback validation method with a proportion of 0.33 and a random seed of 0 was implemented. To gain further insights into the SVM-RBF model's behavior [35] and the relationships between features and predicted genotypes, we generated a prediction profiler, interaction profiles, marginal model plots, and surface plots within JMP17 Pro.

## 3 Results

### 3.1 Correlations between traits in cacao trees

The analysis of correlations uncovered distinct patterns in the relationships between flower traits for both CCN51 and SCA6 genotypes (Figs. S1 and S2). In the CCN51 genotype, we found a moderate negative correlation between vertical height and the number of flowers at developmental stages 58–59 (Pearson correlation coefficient (PCC) = −0.428, *p* = 0.003) (Fig. S1). This indicates that as vertical height increases, the number of flowers at these stages decreases. However, this pattern did not extend to the total flower count across all developmental stages. Additionally, negative correlations were found between vertical height and flower size and shape attributes (area, perimeter, width) at stages 58–59.

Conversely, there was a positive correlation between height and the LWR, a shape-related trait. In the SCA6 genotype, a similar trend of negative correlation between vertical height and flower sizes was observed, with the strength of this association varying based on data aggregation (PCC = −0.21 to −0.54, *p* < 0.05) (Fig. S2). Shape-related traits also exhibited associations with vertical height, with LWR showing a positive correlation and circularity demonstrating a negative correlation. However, one notable difference in SCA6 was that there was no significant correlation between flower number and vertical height, regardless of the analysis methods used.

**Fig. 2:**
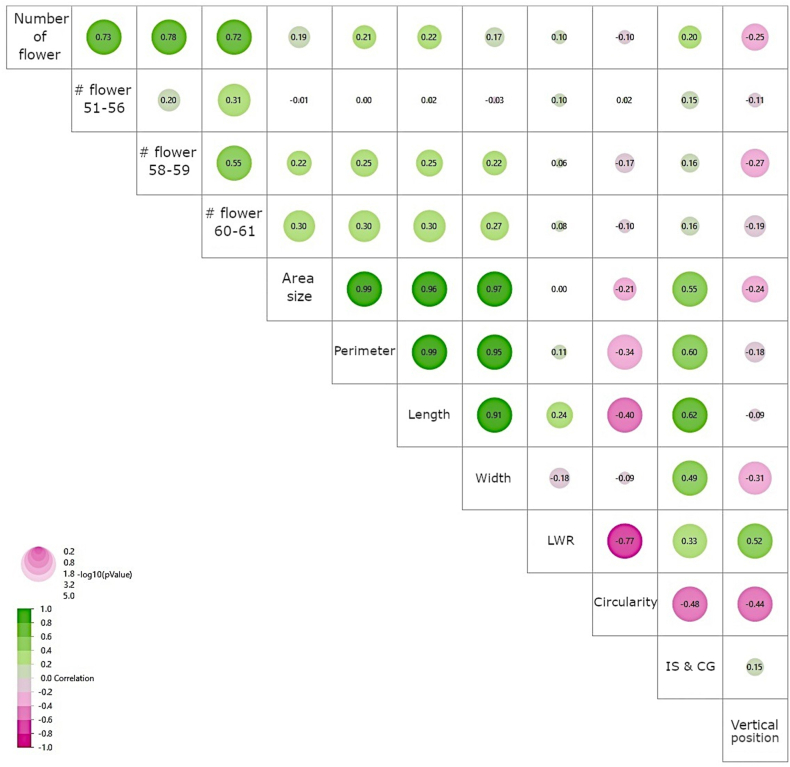
Correlation analysis of flower traits in two cacao genotypes, stratified by vertical height of flower cushions. Pearson correlation coefficients reveal strong interrelationships among most traits in both CCN51 and SCA6 genotypes. Notable exceptions include the number of earlier-stage flowers (growth stage 51–56), which showed no significant correlation with vertical height or flower morphology at stage 58–59. Additionally, the leaf weight ratio (LWR) of flowers at stage 58–59 was not correlated with other flower number-related traits.

When analyzing data combined from both genotypes, we found strong interrelationships among most flower traits, indicating a complex network of interactions governing flower development (Fig. 2). Some exceptions were observed, such as the lack of correlation between the number of early-stage flowers (stages 51–56) and morphological traits of later-stage flowers or vertical height, as well as between LWR at stage 58–59 and flower abundance. Notably, moderate but significant correlations were identified between vertical height and traits like circularity, LWR, width, and the number of flowers (*p* < 0.05), suggesting a subtle influence of vertical position on flower morphology and abundance.

### 3.2 Contrasting phenotypic traits in CCN51 and SCA6

A comparative analysis between the CCN51 and SCA6 genotypes revealed significant differences in key floral traits (Table 1). Specifically, flowers of CCN51 at developmental stages 58–59 exhibited significantly larger dimensions (width, length, perimeter, and area size) than those of SCA6. In contrast, SCA6 flowers at the same developmental stage were more circular. Moreover, CCN51 produced more flowers at growth stages 58–59 and had more per cushion than SCA6. Additionally, CCN51 exhibited a higher number of open flowers and higher values for traits such as LWR and the distance between IS and CG. The vertical distribution of flowers did not differ significantly between the two genotypes.

**Table 1:**
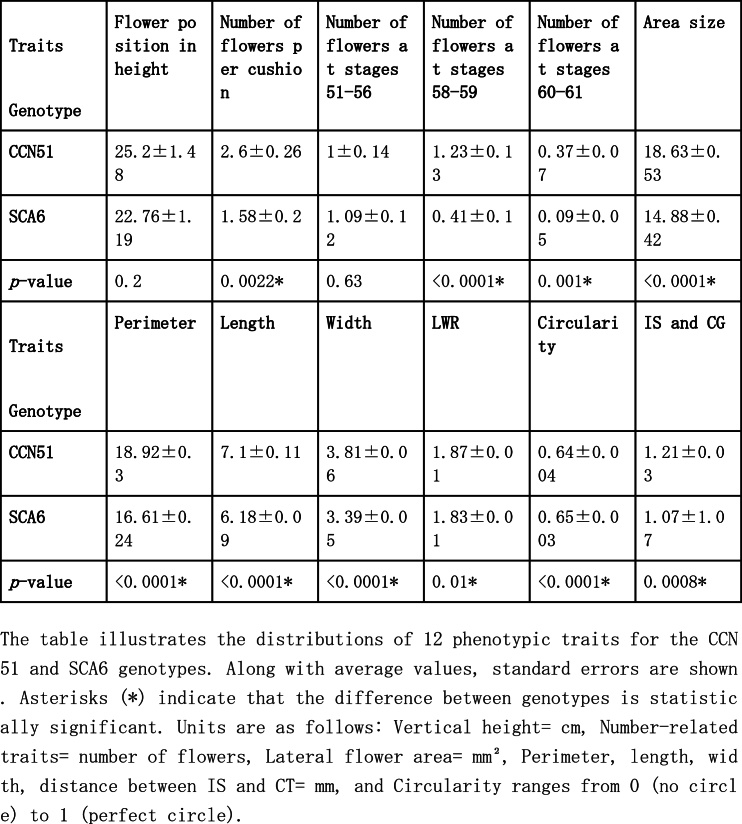
Comparison of Phenotypic Traits between CCN51 and SCA6 Genotypes.

### 3.3 Principal component analysis and hierarchical clustering analysis

**Fig. 3:**
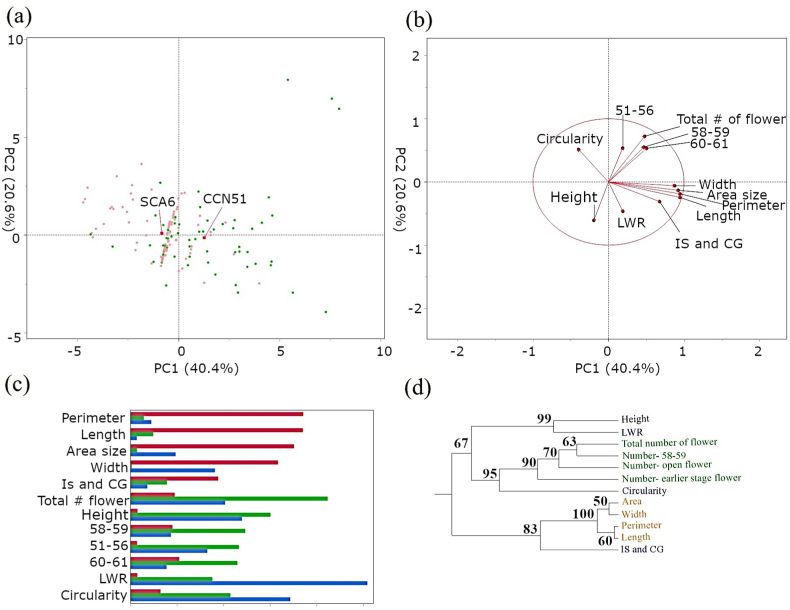
Multivariate Analysis of Cacao Traits and Genotypes (a) PCA biplot illustrating the distribution of two cacao genotypes (SCA6 in pink, CCN51 in green) based on 12 phenotypic traits. The average value for both genotypes is indicated in red. (b) PCA biplot specifically highlighting phenology-related traits in the two cacao genotypes. The two major principal components (PC1 and PC2) explain 61 % of the variance in the data. (c) Bar chart depicting the contribution of each trait to the first three principal components (PC1: red, PC2: blue, PC3: green) in the PCA analysis. (d) Dendrogram showcasing the hierarchical clustering of the 12 phenotypic traits based on their similarity. The clustering analysis reveals distinct groupings of size-related traits (orange) and flower number-related traits (green), as well as a close relationship between height and LWR (length-to-width ratio). Shape-related traits (blue) show a more dispersed distribution across the dendrogram. Numbers on the nodes indicate bootstrap value (% from 1,000 replications).

We utilized PCA to explore the phenotypic diversity and relationships among the tested traits in two cacao genotypes. The PCA biplot (Fig. 3a) visually represents the distribution of CCN51 and SCA6 accessions based on 12 phenotypic traits. Notably, the first two principal components (PCs) accounted for 61 % of the total variance. A separate PCA focusing solely on flower size and shape-related traits revealed that 96 % of the variance was captured by the first two PCs, underscoring the significance of these traits in phenotypic differentiation (data not shown). The average values for both genotypes, marked in red, show a clear separation between CCN51 and SCA6, with CCN51 generally displaying larger flower sizes. A distinct clustering of traits was observed in a focused PCA biplot specifically examining feathered traits, with flower size-related traits forming one cluster and flower number-related traits forming another. Traits such as circularity, vertical position (height) of flowers, and LWR were distinctly separated from other traits (Fig. 3b). Furthermore, Fig. 3c illustrates each trait's contribution to the first three principal components. Size-related traits (area, width, perimeter, and length) exhibited strong loadings on PC1, emphasizing their significance. PC2 was primarily influenced by shape-related traits (circularity, LWR), highlighting their contribution to capturing additional phenotypic variation. PC3 primarily explained flower number-related traits. Additionally, hierarchical clustering analysis (Fig. 3d) supported these findings, revealing distinct groupings of size-related and flower number-related traits, indicating their strong correlations. Meanwhile, shape-related traits displayed a more scattered distribution, with LWR clustering closely with vertical height.

### 3.4 Machine learning analysis

**Fig. 4:**
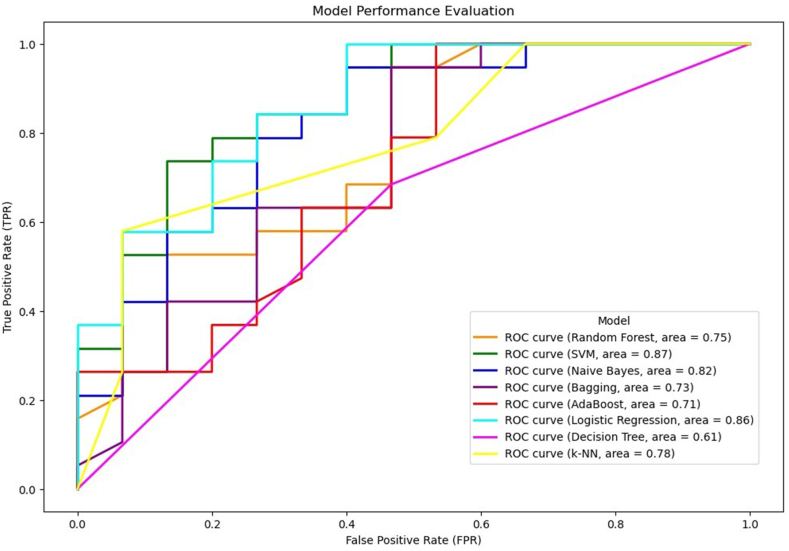
Model performance comparison using ROC curves. ROC curves and AUC values are used to evaluate multiple classification models. SVM exhibits the highest overall performance (AUC = 0.87), followed closely by Logistic Regression and Naive Bayes. Decision Tree lags behind with the lowest AUC (0.61). ROC curves plot the true positive rate against the false positive rate, and the AUC represents the overall ability of the model to discriminate between the two classes. Different color lines indicate different models.

To evaluate the ability of various classification models to distinguish between the two genotypes (CCN51 or SCA6) based on the studied traits (Fig. 4), we conducted a ROC curve analysis. An ROC curve is a graphical plot that illustrates the ability of a binary classifier system. Among the eight models tested, SVM demonstrated the highest overall performance, with an AUC (a measure of the model's ability to correctly classify the genotypes) of 0.87, indicating its strong performance distinguishing between the CCN51 and SCA6 genotypes. Logistic Regression and Naive Bayes followed closely, with AUCs of 0.86 and 0.82, respectively, proving good predictive capabilities. In contrast, the Decision Tree model exhibited the lowest performance with an AUC of 0.61, indicating a less effective separation between the two genotypes. The remaining models, including Random Forest, Bagging, AdaBoost, and k-NN, showed moderate performance, with AUCs ranging from 0.71 to 0.78.

**Fig. 5:**
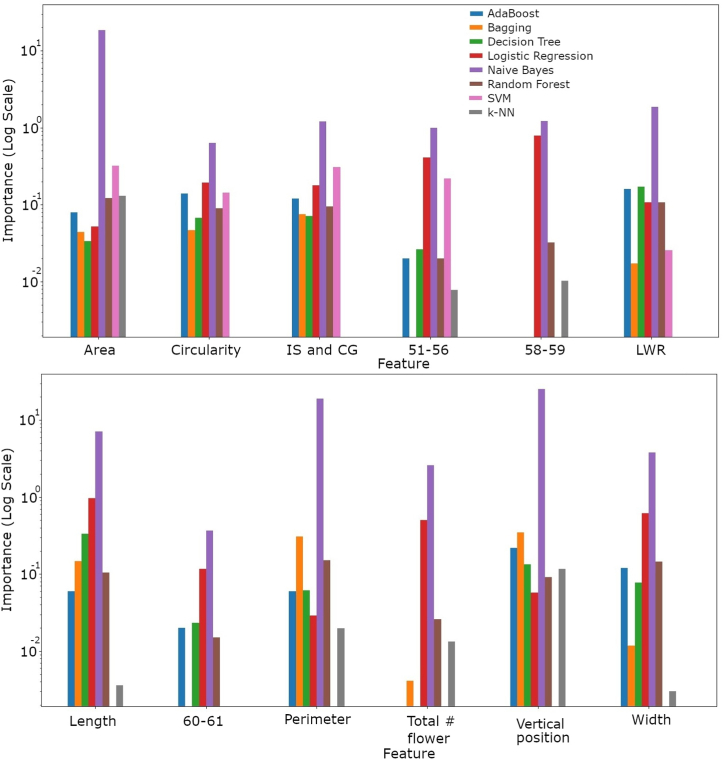
Feature importance comparison across eight machine learning models. This figure illustrates the relative importance of various features in predicting genotypes across eight machine learning models. Different color lines indicate different models.

Fig. 5 illustrates the relative significance of different traits in predicting genotypes across the eight machine learning models. Traits related to flower size, shape, number at various stages, and vertical position consistently influenced most models. However, the models differed in how they weighted specific traits. For example, the Bagging model did not consider the number of flowers at certain stages. In contrast, the SVM model did not incorporate size-related traits (length, width, and perimeter) and the number of flowers at growth stages 58–59, 60–61, and across all stages. On the other hand, logistic regression and Naive Bayes models quite evenly weighed all traits in their predictions. The varying feathered importance across models highlights how different algorithms leverage the available information for genotype prediction.

To further explore potential non-linear relationships and trait interactions, we extended our analysis to include SVM with RBF kernel. This SVM-RBF model outperformed even the linear SVM, achieving an AUC of 0.90 on the training set and 0.72 on the validation set. However, as shown in Table 2, the model's performance varied notably between the two genotypes, exhibiting high accuracy for SCA6 but considerably lower accuracy for CCN51. This discrepancy suggests either potential overfitting to the SCA6 genotype or greater inherent variability in the flower traits of CCN51.

**Table 2:** **SVM-RBF model performance for genotype classification using all 12 features.** High accuracy was achieved for both feature sets in the training dataset. SCA6 was classified with greater accuracy compared to CCN51.

Training set			Validation set		
Actual	Predicted rate		Actual	Predicted rate	
Genotype	CCN51	SCA6	Genotype	CCN51	SCA6
CCN51	0.698	0.302	CCN51	0.591	0.409
SCA6	0.104	0.896	SCA6	0.088	0.912

**Fig. 6:**
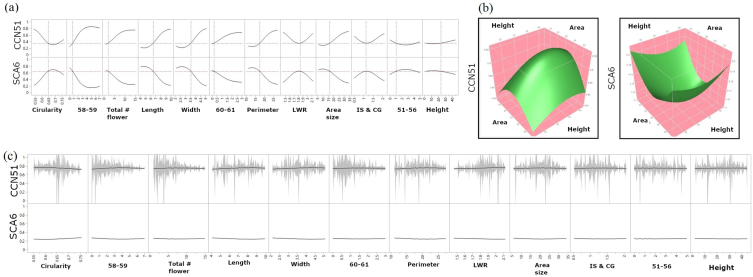
Intricate relationships among the twelve features. (a) Prediction profiler illustrating the non-linear relationships between various flower traits and the predicted genotype probabilities for both CCN51 and SCA6. (b) Surface plots visualizing the complex interaction between vertical height and flower area size at stage 58–59 for CCN51 (left) and SCA6 (right) show non-linear complex relationships. (c) Marginal model plots for both CCN51 (top) and SCA6. The gray areas represent the confidence intervals around the predicted relationships between each predictor variable (x-axis) and the probability of belonging (y-axis) to the respective genotype.

The intricate relationships between flower features and genotype predictions within the SVM-RBF model were visualized (Fig. 6). The prediction profiler (Fig. 6a) underscored the non-linear influence of various flower traits on genotype classification. Surface plots (Fig. 6b) exemplified the complex interplay between vertical height and flower area size, revealing genotype-specific patterns. Marginal model plots (Fig. 6c) further elucidated the individual contributions of each predictor and the model's confidence in its predictions, with confidence intervals visually represented. Additionally, interaction profile plots (Figs. S3 and S4) provided insights into the interdependencies between pairs of traits for both genotypes. Notably, vertical height consistently interacted with LWR in both genotypes. The SVM-RBF model also detected subtle interactions involving area size-related traits and the number of flowers at growth stage 51–56 within a single cushion. This comprehensive machine learning analysis underscores the potential of advanced computational techniques in elucidating the complex relationships between phenotypic traits and genetic backgrounds in cacao.

## 4 Discussion

Our study partially supports the hypothesis that environmental and physiological gradients associated with vertical flower position, in conjunction with genotypic variation, influence flower morphology and abundance in cacao trees. This was evident in the differences in flower size and shape between genotypes and across vertical positions (Table 1, Fig. 2, S1, and S2). These findings align with the results of our machine learning models, which identified various flower traits as significant predictors of genotype, suggesting a strong link between phenotypic traits and underlying genetic differences.

The dendrograms in Fig. 3d revealed an association between vertical height and the LWR, a measure of flower shape, suggesting that vertical position may influence flower morphology. The vertical positioning of flowers affects their access to light and other environmental factors, which can influence the distribution of growth hormones such as auxins, cytokinins, and strigolactones. These hormones play critical roles in bud activation and development, creating physiological gradients that may impact flower shape and other morphological traits [11]. The dispersed distribution of shape-related traits—circularity, LWR, and the distance between the IS and CG—across the dendrogram hints at a complex regulatory network or a degree of independence from factors influencing size and flower number. The comparison of clustering patterns between CCN51 and SCA6 revealed both similarities and differences in trait associations, potentially reflecting genotypic variations in flower development and morphology.

Understanding the influence of vertical position on cacao flower morphology and abundance is crucial for effective cultivation practices. By optimizing canopy management to ensure adequate light penetration to lower flowers while maintaining sufficient shade for overall tree health, farmers can enhance cacao production. This relationship between light availability and flower morphology also emphasizes the importance of biodiversity in agroforestry systems [36]. Studies have shown that increased biodiversity can enhance yields [37]. Therefore, optimizing light distribution within cacao plantations can not only improve yields but also contribute to the ecological sustainability of these systems by promoting biodiversity and reducing the need for artificial inputs [23].

The negative correlation between vertical height and the number of flowers per cushion, particularly pronounced in CCN51 (Table 1), suggests a potential shift in flowering strategy with increasing height. While further investigation is needed to confirm this, the total number of flowers remains relatively stable across the tree's vertical profile; CCN51 appears to compensate for fewer flowers per cushion at higher positions by potentially altering its flower distribution, which may be related to differences in branching patterns. This indicates that CCN51 may control flower distribution vertically by producing more cushions higher up the tree but fewer flowers per cushion. In contrast, in SCA6, the number of flowers was not correlated with vertical height, regardless of whether flower cushions were pooled by height or analyzed individually. This implies that, unlike CCN51, SCA6 does not regulate flower numbers based on vertical position. The difference in flower number regulation by genotype might be attributed to varying light exposure at different tree heights, physiological age, resource allocation strategies, inherent genotypic differences, or combinations of these factors. Adjusting flower distribution in response to vertical light gradients might represent an adaptive trait in CCN51, potentially contributing to its greater reproductive success compared to SCA6, and highlighting the genetic diversity within cacao that influences resource allocation and flowering patterns.

Machine learning offers a powerful approach to unraveling complex patterns in phenotypic data and predicting genotypes, especially when dealing with high-dimensional datasets where traditional statistical methods may fall short. Our exploration into the potential of machine learning for genotype prediction, based on the hypothesis that flower measurements could serve as reliable indicators of genetic identity, yielded promising results. The ROC curve analysis revealed that the SVM model exhibited the highest overall discriminatory power with an AUC of 0.87, followed closely by Logistic Regression and Naive Bayes models with AUCs of 0.86 and 0.82, respectively. The SVM's proficiency in distinguishing between CCN51 and SCA6 genotypes suggests that it effectively captured the underlying patterns in the data, possibly due to its ability to handle high-dimensional spaces and non-linear relationships when using appropriate kernel functions.

Despite its simplicity and the known violation of its conditional independence assumption in many real-world datasets, the Naive Bayes model showed high AUC in our study. This suggests that the model was able to effectively capture the relationships between the flower traits and the genotypes, even if the features are not completely independent. Logistic Regression, as a linear model, suggests that there is a linear relationship between the predictors and the genotype classes. On the other hand, the Decision Tree and k-NN models demonstrated lower AUC values, indicating that their predictive performance was less effective in this context. This could be due to overfitting in the case of Decision Trees or sensitivity to the choice of ‘k' and distance metrics in k-NN.

Analysis of feature importance across the models revealed that most models were influenced by a combination of key flower traits, including size, shape, and flower number at different developmental stages (Fig. 5). However, there were variations in how different algorithms weighted specific features. For instance, some models did not consider certain flower numbers at specific stages or size-related traits. The SVM model's excellent performance, even when certain features were omitted, indicates its capability to identify the most important phenotypic traits for accurately distinguishing between cacao genotypes. These variations in feature importance underscore the complexity of the genotype classification problem and the diverse strategies employed by different algorithms to learn from the data.

To further explore the complex relationships between flower traits and genotype, we employed SVM with RBF kernel. This model achieved an impressive AUC of 0.90 on the training set, highlighting its ability to capture nonlinear interactions within the data. Intriguingly, the SVM-RBF model revealed contrasting classification accuracies between the genotypes. While SCA6 was classified with high accuracy, the accuracy for CCN51 was notably lower. This discrepancy suggests potential overfitting to the SCA6 genotype or greater variability in the phenotypic expression of CCN51, warranting further investigation into the genetic and environmental factors influencing its floral traits.

The visualization tools provided valuable insights into the intricate relationships between flower features and genotype predictions within the SVM-RBF model. The prediction profiler highlighted the non-linear influence of various traits on genotype classification, while surface plots revealed genotype-specific patterns in the interaction between vertical height and flower area size. Interaction profile plots further elucidated the individual and combined contributions of predictors, highlighting the complex interplay of factors influencing genotype differentiation (Figs. S3 and S4). Notably, vertical height consistently interacted with LWR in both genotypes, and subtle interactions were detected between area-related traits and early-stage flower numbers. These results underscore the power of machine learning in identifying key phenotypic traits and accurately predicting cacao genotypes. However, the erratic nature of cacao flower counts, even within the same genotype and vertical position, highlights the complexity of its reproductive biology. This inherent variability, likely driven by micro-environmental factors such as light and resource availability, has important implications for yield prediction, suggesting that caution is needed when relying solely on flower counts as indicators of future production. Applying these machine learning tools to larger datasets encompassing diverse genotypes and environments could revolutionize tropical tree crop breeding by enabling more precise selection for desirable traits, including those that are the product of local adaptation via intense natural selection.

The identification of key floral traits linked to genotype, coupled with machine learning's predictive power, lays the groundwork for early screening of desirable flowering patterns in cacao. This is particularly relevant for speed breeding [38], where rapid generation advancement is essential for research purposes. Integrating these insights with high-throughput phenotyping platforms could enable indirect selection of superior genotypes at the seedling stage, mirroring approaches proposed for other tree species [39]. A recent study highlighted the potential of predictive genomics and machine learning to merge heterogeneous genomic and phenotypic data, which could be adapted for cacao to identify correlations between early-stage traits and mature flowering patterns [39]. For instance, machine learning models trained on seedling image data, potentially capturing traits like leaf morphology or early bud development, might allow rapid, non-destructive assessment of future floral traits, accelerating the selection process.

The observed variation in flowering patterns between genotypes and across vertical positions also raises the intriguing possibility of utilizing rootstock propagation to stabilize or modify these traits in cacao [40]. As demonstrated by several studies, rootstocks can significantly influence scion phenotype, including reproductive traits [[40], [41], [42], [43]]. It is conceivable that selecting rootstocks with specific traits could help to mitigate the erratic nature of flower production or promote more desirable flowering patterns, such as increased flower abundance at lower positions on the tree, potentially enhancing pollination efficiency and yield stability. This approach could involve selecting rootstocks that promote more consistent flower production across different environments or that enhance the expression of desirable flowering traits in the scion variety. Further research into the interaction between rootstock and scion genotypes in cacao is warranted to explore the potential of this approach for improving and stabilizing flowering patterns in seedling orchards and plantations.

Furthermore, future research should investigate the molecular mechanisms underpinning the observed phenotypic differences between CCN51 and SCA6. Transcriptomic and metabolomic profiling could provide valuable insights into the gene expression patterns [44] and metabolic pathways [45] associated with these variations. Additionally, exploring the role of epigenetic modifications in flower development in response to vertical position could elucidate how cacao plants adjust their reproductive strategies under varying environmental conditions. Field studies are also crucial to validate these greenhouse findings and assess their applicability in real-world cacao production systems, considering the influence of diverse environmental factors and the broader range of cacao genotypes in cultivation.

In summary, this study underscores the intricate relationship between vertical flower position, morphology, and genotype in cacao. Our findings demonstrate that vertical position significantly influences flower traits, highlighting the importance of optimizing canopy management and light distribution for improved productivity. Furthermore, the successful application of machine learning for genotype prediction based on floral characteristics opens exciting possibilities for accelerating cacao breeding and selection programs. Through the integration of phenotypic analysis & machine learning and forthcoming molecular and field studies, there is potential to advance our comprehension of cacao reproductive biology. This study contributes to a critical understanding of cacao biology needed to address the challenges of climate change [46]. This knowledge can aid in developing cacao varieties with improved resilience to changing climatic conditions, such as drought or increased temperatures, and contribute to the long-term sustainability of cacao production.

## CRediT authorship contribution statement

**Seunghyun Lim:** Writing – review & editing, Validation, Software, Formal analysis. **Insuck Baek:** Writing – review & editing, Validation, Software, Formal analysis. **Seok Min Hong:** Writing – review & editing, Validation, Software, Formal analysis. **Yoonjung Lee:** Writing – review & editing, Methodology. **Silvas Kirubakaran:** Writing – review & editing, Methodology. **Moon S. Kim:** Writing – review & editing, Supervision, Resources, Funding acquisition. **Lyndel W. Meinhardt:** Writing – review & editing, Supervision, Resources, Funding acquisition. **Sunchung Park:** Writing – review & editing, Methodology. **Ezekiel Ahn:** Writing – original draft, Supervision, Project administration, Methodology, Investigation, Funding acquisition, Formal analysis, Data curation, Conceptualization.

## Informed consent statement

Not applicable.

## Institutional review board statement

Not applicable.

## Data availability statement

Not available.

## Funding

S.L., I.B., M.S.K., L.W.M., S.P., and E.A. were supported by the U.S. Department of Agriculture, Agricultural Research Service, through In-House Projects No. 8042-21220-258-000-D and 8042-21000-303-000-D. Mention of any trade names or commercial products in this article is solely for the purpose of providing specific information and does not imply recommendation or endorsement by the U. S. Department of Agriculture. USDA is an equal opportunity provider and employer, and all agency services are available without discrimination.

## Declaration of competing interest

The authors declare no competing interests.

## Figures and Tables

**Figure:**
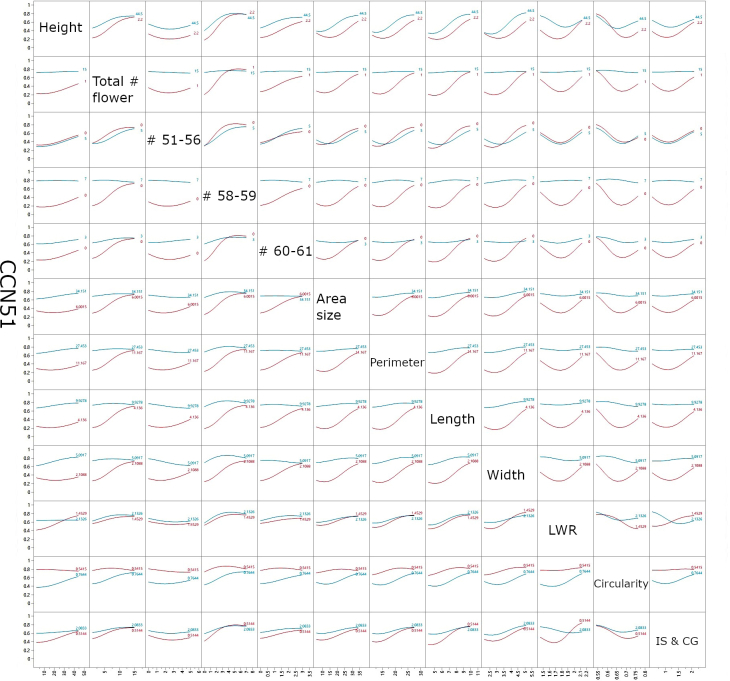


**Figure:**