# Supplementary material for: Cacao floral traits are shaped by the interaction of flower position with genotype
Source: Heliyon. 2025 Feb 3;11(4):e42407. doi: 10.1016/j.heliyon.2025.e42407 (PMC11867291; doi:10.1016/j.heliyon.2025.e42407)
Supplement: Multimedia component 2 [file mmc2.pdf]

CellPress

CELL PRESS DECLARATION OF INTERESTS POLICY

Transparency is essential for a reader's trust in the scientific process and for the credibility of published articles. At Cell Press, we feel that disclosure of competing interests is a critical aspect of transparency. Therefore, we require a "declaration of interests" section in which all authors disclose any financial or other interests related to the submitted work that (1) could affect or have the perception of affecting the author's objectivity or (2) could influence or have the perception of influencing the content of the article.

What types of articles does this apply to?

We require that you disclose competing interests for all submitted content by completing and submitting the form below. We also require that you include a "declaration of interests" section in the text of all articles even if there are no interests to declare.

What should I disclose?

We require that you and all authors disclose any personal financial interests (e.g., stocks or shares in companies with interests related to the submitted work or consulting fees from companies that could have interests related to the work), professional affiliations, advisory positions, board memberships (including membership on a journal's advisory board when publishing in that journal), or patent applications and/or registrations that are related to the subject matter of the contribution. As a guideline, you need to declare an interest for (1) any affiliation associated with a payment or financial benefit exceeding $10,000 p.a. or 5% ownership of a company or (2) research funding by a company with related interests. You do not need to disclose diversified mutual funds, 401ks, or investment trusts.

Authors should also disclose relevant financial interests of immediate family members. Cell Press uses the Public Health Service definition of "immediate family member," which includes spouse and dependent children.

Where do I declare competing interests?

Competing interests should be disclosed on this form as well as in a "declaration of interests" section in the manuscript. This section should include financial or other competing interests as well as affiliations that are not included in the author list. Examples of "declaration of interests" language include:

> "AUTHOR is an employee and shareholder of COMPANY."
> "AUTHOR is a founder of COMPANY and a member of its scientific advisory board."

NOTE: Primary affiliations should be included with the author list and do not need to be included in the "declaration of interests" section. Funding sources should be included in the "acknowledgments" section and also do not need to be included in the "declaration of interests" section. (A small number of front-matter article types do not include an "acknowledgments" section. For these articles, reporting of funding sources is not required.)

What if there are no competing interests to declare?

If you have no competing interests to declare, please note that in the "declaration of interests" section with the following wording:

> "The authors declare no competing interests."

April 12, 2024

CELL PRESS DECLARATION OF INTERESTS FORM

If submitting materials via Editorial Manager, please complete this form and upload with your initial submission. Otherwise, please email as an attachment to the editor handling your manuscript.

Please complete each section of the form and insert any necessary "declaration of interests" statement in the text box at the end of the form. A matching statement should be included in a "declaration of interests" section in the manuscript.

Institutional affiliations

We require that you list the current institutional affiliations of all authors, including academic, corporate, and industrial, on the title page of the manuscript. ***Please select one of the following:***

- ☒ All affiliations are listed on the title page of the manuscript.
- ☐ I or other authors have additional affiliations that we have noted in the "declaration of interests" section of the manuscript and on this form below.

Funding sources

We require that you disclose all funding sources for the research described in this work. ***Please confirm the following:***

- ☒ All funding sources for this study are listed in the "acknowledgments" section of the manuscript.*

*A small number of front-matter article types do not include an "acknowledgments" section. For these, reporting funding sources is not required.

Competing financial interests

We require that authors disclose any financial interests and any such interests of immediate family members, including financial holdings, professional affiliations, advisory positions, board memberships, receipt of consulting fees, etc., that:

(1) could affect or have the perception of affecting the author's objectivity, *or*
(2) could influence or have the perception of influencing the content of the article.

Please select one of the following:

- ☒ We, the authors and our immediate family members, have no financial interests to declare.
- ☐ We, the authors, have noted any financial interests in the "declaration of interests" section of the manuscript and on this form below, and we have noted interests of our immediate family members.

<u>Advisory/management and consulting positions</u>

We require that authors disclose any position, be it a member of a board or advisory committee or a paid consultant, that they have been involved with that is related to this study. We also require that members of our journal advisory boards disclose their position when publishing in that journal. ***Please select one of the following:***

☒ We, the authors and our immediate family members, have no positions to declare and are not members of the journal's advisory board.

☐ The authors and/or their immediate family members have management/advisory or consulting relationships noted in the "declaration of interests" section of the manuscript and on this form below.

<u>Patents</u>

We require that you disclose any patent applications and/or registrations related to this work by any of the authors or their institutions. ***Please select one of the following:***

☒ We, the authors and our immediate family members, have no related patent applications or registrations to declare.

☐ We, the authors, have a patent application and/or registration related to this work, which is noted in the "declaration of interests" section of the manuscript and on this form below, and we have noted the patents of immediate family members.

Please insert any "declaration of interests" statements in this space. This exact text should also be included in the "declaration of interests" section of the manuscript. If no authors have a competing interest, please insert the text, "The authors declare no competing interests."

The authors declare no competing interests.

☒ **On behalf of all authors, I declare that I have disclosed all competing interests related to this work. If any exist, they have been included in the "declaration of interests" section of the manuscript.**
